# Smartphone-based fundus imaging for evaluation of Retinopathy of Prematurity in a low-income country: A pilot study

**DOI:** 10.12669/pjms.39.3.7053

**Published:** 2023

**Authors:** Roha Ahmad Choudhary, Shiraz Hashmi, Haroon Tayyab

**Affiliations:** 1 Roha Ahmad Choudhary, MBBS. Department of Ophthalmology and Visual Sciences, The Aga Khan University Hospital, Karachi Stadium, Stadium Road, Karachi, Pakistani; 2 Shiraz Hashmi, MBBS, MPH. Department of Ophthalmology and Visual Sciences, The Aga Khan University Hospital, Karachi Stadium, Stadium Road, Karachi, Pakistani; 3 Haroon Tayyab, MBBS, FCPS (Ophth), FCPS (VRO), FRCS (Glasg), FACS. Department of Ophthalmology and Visual Sciences, The Aga Khan University Hospital, Karachi Stadium, Stadium Road, Karachi, Pakistani

**Keywords:** Retinopathy of prematurity, Plus disease, Indirect ophthalmoscope

## Abstract

**Objectives:**

To evaluate the feasibility of a novel and simple smart phone-based Retinopathy of Prematurity (ROP) screening approach in a resource-constrained setting.

**Methods:**

This cross-sectional validation study was conducted at the Department of Ophthalmology and Neonatal Intensive Care Unit (NICU) of The Aga Khan University Hospital, Pakistan, from January 2022 to April 2022. A total of 63 images of eyes with active ROP (stage-1, 2, 3, 4 and/or plus or pre-plus disease) were included in this study. The stage of ROP was documented by the principal investigator using an indirect ophthalmoscope and retinal images were obtained using this novel technique. These images were shared with two masked ROP experts who rated the image quality and determined the stage of ROP and presence of plus disease. Their reports were compared with the initial findings reported by principal investigator using indirect ophthalmoscope.

**Results:**

We reviewed 63 images for image quality, stage of ROP and presence of plus disease. There was significant agreement between the gold standard and the Rater-1 and 2 for the presence of plus disease (Cohen’s kappa was 0.84 and 1.0) and the stage of the disease (Cohen’s kappa 0.65 and 1.0). There was significant agreement between the Rater for presence of plus disease and any stage of ROP (Cohen’s κ: 0.84 and 0.65 for plus disease and any stage of the ROP, respectively). Rater-1 and 2 rated 96.83% and 98.41% images as excellent / acceptable respectively.

**Conclusions:**

High quality retinal images can be captured with a smartphone and 28D lens without using any additional adapter equipment. This approach of ROP screening can form basis of telemedicine for ROP in resource constrained areas.

## INTRODUCTION

Retinopathy of prematurity (ROP) is one of the leading causes of preventable pediatric blindness worldwide. First-world countries have developed adequate strategies to reduce the burden of childhood blindness through vastly improved ophthalmic and neonatal screening and treatment protocols. In total, 52.9% of global premature births have been reported to occur in Asia, with countries such as Pakistan among the top ten in the world, with an estimated 3.0% share of global preterm births in 2014.^1^ Another report suggests that 40% of preterm births in the world occur in five countries (India, Pakistan, Indonesia, China, Bangladesh). This high rate of preterms in this region is labeled the ’third epidemic’ of ROP.^2^

This ‘third epidemic’ is mainly attributed to the enhanced neonatal survival efforts in the Low Middle Income Countries (LMICs) but poor concomitant focus on debilitating complications such as ROP that can occur in the premature infants. One of the reasons is the inadequate screening of ROP. This is due to the lack of trained ROP specialists, the difficult access of the population to resource centers, and all the suboptimal level of awareness among health care providers.^3^ With this as background, we are not expecting the current pool of ROP specialists to meet the ever exploding need of ROP screening in LMICs. There is an emerging need to revise the way we approach ROP. We have to develop novel ROP screening strategies which are efficient, effective, cost friendly with high level of sensitivity and specificity.^4^

Telemedicine in ROP has been regularly utilized to overcome the scarcity of trained ROP specialists and many models have been effective as well. With this approach comes a significant cost of installing high-cost retinal imaging hardware in LMICs where ROP specialists are not available. Also, this requires training of non-physician personnel to capture retinal images using these cameras.^5^ This can pose a significant barrier to ROP telemedicine in LMICs. Hashmi et al. reported that even in Karachi, the largest and most populous city of the Pakistan, a clear lack of understanding of ROP screening and referral protocol was found in physicians caring for premature infant.

They also found out that majority of the physicians did not know about the sight-threatening nature of the disease and only two of the surveyed centres arranged neonate examinations by an ophthalmologist albeit not in accordance with international guidelines.^6^ This may change due to recent improvements in smart phone camera technology and its wide spread use. There have been reports in which the use of smart phone-assisted retinal imaging has been used successfully and comparisons have been made between indirect ophthalmoscopes and images taken by smart phones.^4^,^7^

So far, smart phone-based retinal imaging in ROP has been assisted with the use of an adapter to hold a lens or/and a smart phone. There are many types of adapter available for this purpose and at times, these adapter can be smart phone model specific which may not be a long term reliable solution. In our study, we describe a novel technique to acquired retinal images in babies with ROP using smart phone and 28D lens without the need of any additional equipment.

The rationale for this study is that the smart phone camera can capture highly clear and informative retinal images and can be used for telemedicine for ROP cases in LMICs. This is a very cost effective and readily available method to combat the mounting challenge of ROP screening in Pakistan. We believe that in the future, a similar model can be adopted in our setting by training technicians or even pediatric physicians in hospitals with no eye specialists to capture high-quality fundus imaging via the smartphone technique we describe.

These fundus images can then be shared with specialists via an online database for accurate evaluation and management and involve much lower costs since no additional equipment is needed for imaging or record management in our technique. This also helps parents get actively involved in the care as counselling can be done through direct evidence of pathology though the retinal images. To our knowledge, this the first study where smart phone is being used to pilot a strategy for ROP telemedicine in Pakistan which is one of the five countries where burden of ROP is the most and resources are very limited.

## METHODS

This is a cross-sectional validation study conducted in the Department of Ophthalmology and Neonatal Intensive Care Unit (NICU) of The Aga Khan University Hospital, Pakistan, from January 2022 to April 2022. Parents of all babies were explained about the retinal imaging and a written and informed consent was obtained for all patients. We sought approval for this study from Ethical Review Board of the hospital (ERC # 2022-7036-20602). During this study, we adhered to the principles of the Declaration of Helsinki.

This study used purposive sampling where we included premature babies in NICU and those presenting in outpatient clinic for ROP screening. All babies who had some stage of ROP (Stage-0, 1,2,3,4 or plus/pre-plus disease) were included in this study. This inclusion criteria was used to demonstrate the ability of smart phone based retinal imaging as an efficient tool to document and report (in future, telemedicine use) patients with active ROP. Since the masked readers were expert in ROP examination, we did not need normal retinal images to validate the tool. All babies who had undergone interventions (intravitreal injection, laser) were not included in this study.

Eyes of all babies were dilated using phenylephrine 0.5%, tropicamide 1%, and proparacaine 0.5%. A nurse monitored heart and respiratory rate, temperature, and oxygen saturation during entire examination. The primary investigator (HT) recorded this assessment using the gold standard indirect ophthalmoscope. The eyes of babies were held open with a wire speculum and an assistant gently stabilized the head of babies. A drop of basic salt solution was used to avoid cornel dryness during examination. Then, video of the fundus was captured using a smartphone of the same model and brand for all the study participants (iPhone 11, Apple Inc, Cupertino, CA, USA).

The smartphone was held in one hand while a 28 D lens (Volk Optical Inc., Mentor, Ohio, USA) (53 degrees field of view) was held in the other and the distance between the two was adjusted to bring the retina into focus. The phone’s flashlight in video mode was used as illumination source. Attempt was made to capture as peripheral view of retina as allowed with the aim to capture the transition zone. This was made possible by maneuvering the smart phone, just as we maneuver an indirect ophthalmoscope. Videos with 1x magnification were captured from various angles to get maximum information about retina. No indentation was used. Images were extracted from the recorded view and shared with experts (Rater-1: AH: Rater-2: VKA) in a secure and anonymous manner. The Rater recorded the stage of ROP, the status of plus, pre-plus, or no plus disease, and the quality of the image (excellent, acceptable, not gradable).

Data from the primary investigator (HT) and Rater were compiled by a second investigator (RC) on a single Microsoft Excel (Version 2016) worksheet. Statistical analysis was performed using SPSS version 21 (IBM SPSS Statistics for Windows, Version 21.0. Armonk, NY: IBM Corp). Intraclass correlation was calculated to assess inter-reviewer agreement using Cohen’s kappa coefficient (95% confidence interval to correct the effect of chance). The following scale was used to interpret Cohen’s kappa scores: 0.21 to 0.40: fair agreement; 0.41 to 0.60: moderate agreement; 0.61 to 0.80: substantial agreement; and 0.81 to 1.0: near-perfect agreement.

## RESULTS

This study was carried out to assess the ability of the smart phone camera to capture retinal images of preterm infants that are of diagnostic significance. In this study, we captured 63 images (images were extracted from a video recording) that showed varying stages of ROP with plus, pre plus or no plus disease. Out of 63 images, we captured the junction between the vascularized and avascular retina in 43 images. In the remaining 20 patients, we only assessed the status of plus, pre plus or no plus disease. Table-I shows the percentage distribution of various stages of ROP in these 43 images recorded with an indirect ophthalmoscope as the gold standard.

**Table-I:** n=43; Percentage of different stages of ROP.

Stage of ROP	n	%
Stage 0	2	4.65
Stage 1	10	23.25
Stage 2	20	46.51
Stage 3	9	20.93
Stage 4	2	4.65

Total	43	100

**Table-II:** n=63; Percentage of plus, pre plus and no plus disease.

Status	n	%
Plus disease	35	55.55
Pre-plus disease	24	38.09
No Plus disease	4	6.34

Total	63	100

Cohen’s kappa statistics was used to calculate the Rater agreeability with gold standard and between Rater as well. For the presence of plus/pre plus disease, Cohen’s kappa was 0.84 and 1.0 for Rater-1 and 2 when compared to the findings of the indirect ophthalmoscope (gold standard). There was high agreeability when correctly detecting any stage of ROP as compared to gold standard (Cohen’s κ 0.65 and 1.0 for Rater-1 and 2). We also calculated the agreement between the Rater-s for plus disease and the stage of the ROP where we showed a high degree of agreeability (Cohen’s κ: 0.84 and 0.65 for plus disease and any stage of the ROP) in our study.

**Fig.1:**
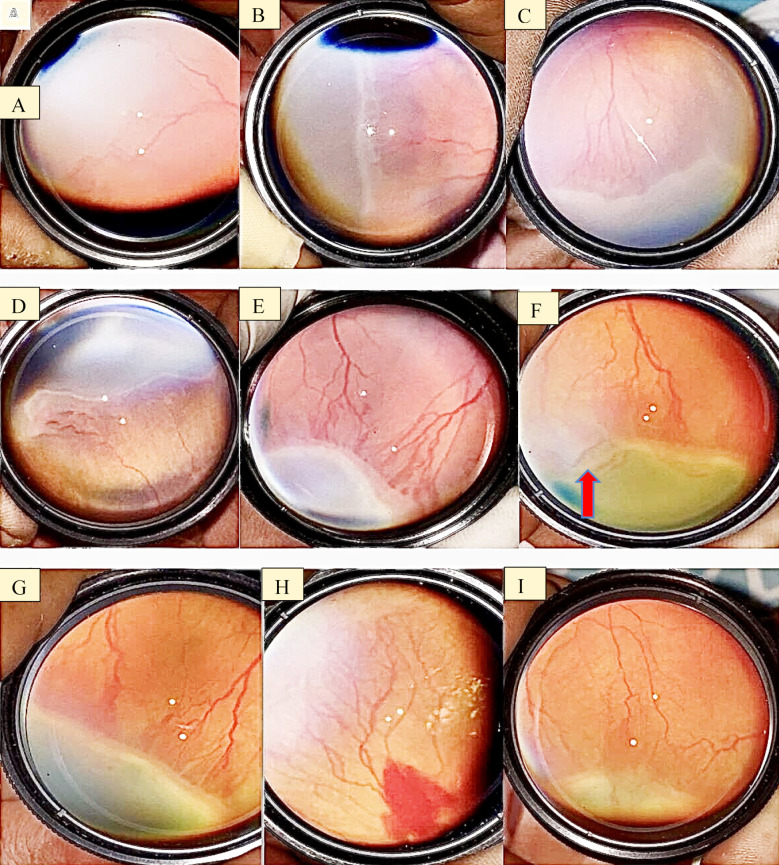
Images captured with smart phone and 28D lens without adapter. **A:** Demarcation line, **B, C and D:** Demarcation ridge, **E, G and I:** Vascularized ridge with dilated and tortuous vessels, **H:** Retinal hemorrhage with dilated and tortuous vessels, **F:** Retinal elevation (red arrow)

Also, similar assessment for image quality where Rater-1 recorded 3.17% as unacceptable and 96.83% as excellent/acceptable images. Rater-2 recorded 1.58% as unacceptable and 98.41% as excellent images. The mean ± standard deviation time to capture a single adequate quality video was 86.61 ± 16.32 seconds. The unacceptable images were not included in final analysis. There were no adverse events during the examination process (tachycardia, bradycardia, apnea, decreased oxygen saturation). Fig.1 shows images taken with the smartphone camera and the 28D lens without any additional devices.

## DISCUSSION

Retinopathy of prematurity was described as retrolental fibroplasia in the 1940s and since then there has been a continuing increase in our understanding of pathogenesis, risk factors and treatment modalities.^8^ Despite all this development, ROP still remains as the leading cause of unnecessary blindness globally.^9^ One of the leading reasons for this unmet need is the limitation in screening high-risk premature babies. This challenge is more accentuated in LMICs where adequate expertise and availability of imaging equipment is scarce.^10^ The challenge of suitable expertise has been tackled using ROP telemedicine in various centers across the globe in last two decades, but the need of routine eye examination was still considered a necessity.

We have worked with a smart phone Volk 28D lens to overcome the challenge of the inaccessibility of highly priced neonatal retinal imaging cameras including RetCam (Natus Medical, Inc, CA, United States) and Phoenix ICON (Phoenix-Micron, Inc, OR, United States). This combination of smart phone and ophthalmic lens was not coupled with any other device or a holder. To our knowledge, this is the first study of its type to use a handheld smart phone and a lens without any additional device to image a premature retina and evaluate the ability to capture ROP.

Various studies have been conducted with varying sample size (43-1257) and reporting sensitivities ranging from 57% to 100% with 95% confidence interval. One such case study, Stanford University for Diagnosis of ROP (SUNDROP) study where NICU nurses used ROP telemedicine to upload images for remote reading. Even with successful models of ROP telemedicine, the lack of very expensive equipment needed to image the neonatal retina remains a widely accepted challenge. Furthermore, the need to train non-physicians to operate complex imaging equipment poses a significant obstacle to the effectiveness of telemedicine ROP.^11^

In recent years, there have been reports in which portable devices have been used effectively to detect and diagnose various retinal pathologies (diabetic retinopathy, retinal vascular disorders, retinal detachment, and optic disc abnormalities). Some have used smart phones coupled with artificial intelligence software to predict stages, whereas others have used it for telemedicine.^12^-^14^ There have been studies in which different smartphone-based software have been tested for better retinal images compared to native camera installed on the phone.^15^

A similar study by Patel et al. utilized a portable plastic 3D printed smart phone based retinal camera (Retina Scope) to evaluate its ability to capture images of diagnostic value for plus disease in cases of ROP and compared with indirect ophthalmoscopy. They demonstrated a high level of inter Rater- agreeability (Cohen’s κ) of two Rater-s who read images from cameras based on the smart phone versus indirect ophthalmoscopy (gold standard) for plus disease (Cohen’s κ: 1.0 and 0.85). These results are similar to our study, in which mutual agreement compared to the gold standard was excellent (Cohen’s κ: 0.84 and 1.0 for Rater-1 and 2) for plus disease detection.

Furthermore, the results of our study are comparable to those of Patel et al. (Cohen’s κ 0.92) when correctly detecting any stage of ROP compared to the gold standard (Cohen’s κ 0.65 and 1.0 for Rater-1 and 2). We also calculated the agreement between the Rater for plus disease and the stage of the ROP where we showed a high degree of agreeability (Cohen’s κ: 0.84 and 0.65 for plus disease and any stage of the ROP) in our study. We also demonstrated a similar assessment for image quality, where Rater-1 recorded 3.17% as unacceptable and 96.83 as excellent/acceptable images. Rater-2 recorded 1.58% as unacceptable and 98.41% as excellent images. These results are similar to the ones recorded by Patel et al.^16^

In one of the earlier studies by Raufi et al, they used Pictor (a handheld non-contact camera by Volk Optical, OH, United States) to evaluate the diagnostic accuracy of Pictor versus video indirect ophthalmoscope (VIO) in evaluating their ability to diagnose plus and pre-plus disease in 23 infants. They concluded that traceability was better with Pictor compared to VIO (73% vs. 98%; 95% CI). The ROC curves also showed better diagnostic accuracy for plus and pre-plus disease with Pictor compared to VIO. This establishes the better performance of the handheld Pictor versus VIO.^17^ Although much less costly than RetCam and Phoenix, it still carries a significant price tag.

A similar study by Lin et al. including 142 eyes of 71 babies showed that retinal images based on smart phones in ROP were acceptable/excellent in 91.4% of examinations, which is also similar to our results.^18^ We also reviewed the results of Sharma et al. where their group used a hand-held mold (to hold a smart phone and a 20D lens in a fixed position and distance from each other) and compared the results with indirect ophthalmoscopy as the gold standard. Cohen’s κ as a measure of interRater- agreement was substantial (κ = 1.0) however it was strong between Rater-1 and Rater-2 (κ=0.84). These results are comparable to our results. The major difference is of the use of holding device for smart phone and lens in Sharma’s study.^19^

Our study adds to the growing evidence that smart phone-based imaging for ROP screening and detection is proving its utility. Although, the field of view captured by smart phone is only 30 degrees as compared to 130 degrees of RetCam, the information gathered through smart phone was significant and vital for accurate diagnosis. In addition, in our study, we did not use any adapter to capture the retinal image as opposed to other studies and this innovation is the first of its nature to be reported in literature. This essentially evades any hardware procurement and costs associated with it and makes ROP screening very cost effective. As opposed to Sharma et al. and Patel et al., our technique was able to capture the transition zone between avascular and vascularized retina which assisted in recording the stage of ROP. One reason was that we used 28D lens whereas 20D lens was used in study by Sharma et al. Patel et al. used the montaging technique to visualize additional retina.^17^-^19^

The strength of our study was its cross-sectional design with no retrospective analysis of any variable, reasonable sample size, double masking between observer and Rater, and single ophthalmologist performing and grading ROP on indirect ophthalmoscopy as the gold standard. Additional strengths include no use of any smart phone or lens holder and our ability to capture the stage of disease despite working with roughly 30-degree field of view.

### Limitations:

The biggest weakness of this study was ophthalmologist taking retinal images through smart phone. Thus, we cannot validate this technique for telemedicine (where images can be captured by non-ophthalmologists, nurses, and technicians). In addition, we have not highlighted the learning curve for taking such sharp and peripheral retinal images, which can take a few weeks of regular training to master. We recommend initial training on suitable and willing adult volunteers in a supine position and then transferring to newborns.

We do not expect increase in the number of ophthalmologists in response to the increasing challenge of ROP. This is linked to multiple factors, including high liability, low reimbursement, and the need for additional training to adequately detect ROP.^20^ There is an overwhelming need of affordable, reliable, effective, and efficient screening and treatment of ROP.

A timely-screened neonate has high chances of avoiding blindness. In addition to this work, we need automated image analysis through artificial intelligence (AI), connected care givers, and effective telemedicine models. One such work is from Brown et al., where they demonstrated comparable and better proficiency at detecting plus disease than ROP experts. Integration of such validated algorithms with smart phones can improve the quality, accessibility, and consistency of ROP screening and reducing the economic burden at the same time.^21^

## CONCLUSION

This is the first study to compare different features of ROP (stage, plus disease) by using a smart phone and lens without holder device or the need of additional equipment. Further work needs to be done to couple similar work with a dedicated smart phone application for control of camera parameters, storage, cataloging and integration with AI algorithms.

### Authors’ contribution:

**RAC:** Did editing of manuscript and bibliography.

**SH:** Performed statistical analysis.

**HT:** Conceived, designed, and prepared the manuscript. He is also responsible for the integrity and accuracy of the study.
